# Evaluation of Online Patient Portal vs Text-Based Blood Pressure Monitoring Among Black Patients With Medicaid and Medicare Insurance Who Have Hypertension and Cardiovascular Disease

**DOI:** 10.1001/jamanetworkopen.2021.44255

**Published:** 2022-02-15

**Authors:** Lauren A. Eberly, Monika Sanghavi, Howard M. Julien, Laura Burger, Neel Chokshi, Jennifer Lewey

**Affiliations:** 1 Division of Cardiovascular Medicine, Perelman School of Medicine, University of Pennsylvania, Philadelphia; 2 Center for Cardiovascular Outcomes, Quality, and Evaluative Research, University of Pennsylvania, Philadelphia; 3 Penn Cardiovascular Center for Health Equity and Social Justice, University of Pennsylvania, Philadelphia; 4 Leonard Davis Institute of Health Economics, University of Pennsylvania, Philadelphia; 5 Penn Center for Digital Cardiology, University of Pennsylvania, Philadelphia

## Abstract

This pilot randomized clinical trial evaluates the increased uptake and acceptability of a text-based model for home blood pressure monitoring compared with online portal use among Black patients with Medicaid and Medicare insurance who have hypertension and cardiovascular disease.

## Introduction

There is a disproportionate burden of uncontrolled hypertension and cardiovascular disease (CVD) among Black patients. Self-measured blood pressure (BP) is associated with improved BP control, especially when combined with telemonitoring.^1^ However, disparities in telemedicine access may limit the benefit of home BP monitoring for Black patients.^2^ The goal of this study was to evaluate the uptake and acceptability of a text-based model for home BP monitoring compared with an online patient portal among Black patients with Medicaid or Medicare insurance who have hypertension and CVD or CVD risk factors.

## Methods

This pilot randomized clinical trial qualified as quality improvement by the University of Pennsylvania Institutional Review Board. Oral consent was obtained from all participants. This trial was registered retrospectively because it was a small feasibility trial; the trial protocol appears in Supplement 1. The study followed the Consolidated Standards of Reporting Trials (CONSORT) reporting guideline for randomized pilot and feasibility trials.

We performed a pilot randomized trial of 20 Black patients who had Medicaid or Medicare insurance, were seen in person for a cardiology consultation, and had hypertension and CVD or 1 or more CVD risk factors based on *International Statistical Classification of Diseases and Related Health Problems, Tenth Revision* diagnosis codes. Race was determined by self-report through the electronic health record. Patients were excluded from this study if they did not speak English, had a BP cuff, or did not have internet access or a phone with texting capabilities.

Patients were randomly assigned 1 to 1 to the text-based group or online patient portal group (standard of care) from November 2020 to February 2021. The health system portal is an internet-based portal that can be accessed via a smartphone app or web browser. All patients were given an automatic upper-arm BP cuff (Omron) and instructions on how to check BP during an in-person visit. Patients were instructed to check their BP twice daily for 14 days. Patients in the text-based group received daily text reminders and an automated text reply based on their BP measurement, with measurements sent to providers at the end of the study period. Patients in the online patient portal group were given instructions on how to enroll in the portal and upload their measurements. They could upload their measurements daily or at the end of 14 days. All patients were given an additional 14 days to send measurements, for a total follow-up of 28 days. After the study period, all participants were sent a text message with a link to a patient survey.

We compared baseline demographic characteristics between groups by using *t* and χ^2^ tests for continuous and categorical variables, respectively. The primary study outcomes were the number of BP measurements and the proportion of patients submitting 1 or more measurements. *P* values < .05 were considered statistically significant. Statistical analyses were performed using SAS version 9.4 (SAS Institute Inc).

## Results

Of the 20 patients enrolled in this study, 10 (50.0%) were women and 13 (65.0%) had Medicaid insurance. The mean (SD) patient age was 55 (10.1) years. Baseline characteristics and outcomes are shown in Table 1. Significantly more patients in the text-based group sent 1 or more BP measurements (10 [100.0%] vs 3 [30.0%]; *P* = .001). The mean (SD) number of BP measurements was 20.9 (10.5) for the text-based group vs 3.9 (6.6) for the online patient portal group (*P* < .001).

**Table 1.** Baseline Characteristics of the Text-Based Message Group and Online Patient Portal Group

Characteristic	No. of patients (%)		*P* value
	Text-based message group (n = 10)	Online patient portal group (n = 10)	
Age, y^a^	53.0 (9.6)	57.7 (10.7)	.31
Sex			
Female	4 (40.0)	6 (60.0)	.37
Male	6 (60.0)	4 (40.0)	
Insurance			.20
Medicaid	8 (80.0)	5 (50.0)	
Medicare	2 (20.0)	5 (50.0)	
Comorbidity			
Obesity	8 (80.0)	9 (90.0)	.53
Coronary artery disease	2 (20.0)	3 (30.0)	.60
Hyperlipidemia	5 (50.0)	8 (80.0)	.16
Diabetes mellitus type 2	4 (40.0)	5 (50.0)	.65
Ischemic cardiomyopathy	2 (20.0)	1 (10.0)	.53
Nonischemic cardiomyopathy	2 (20.0)	0	.14
HFrEF	3 (30.0)	1 (10.0)	.26
HFpEF	1 (10.0)	0	.30
Atrial fibrillation	1 (10.0)	1 (10.0)	>.99
Other arrhythmias	2 (20.0)	1 (10.0)	.53
Cerebrovascular accident	2 (20.0)	1 (10.0)	.53
Chronic kidney disease	3 (30.0)	3 (30.0)	>.99
Prior online portal activation	7 (70.0)	7 (70.0)	>.99
BP at enrollment, mm Hg^a^			
Systolic	131 (14.8)	125 (5.3)	.51
Diastolic	84.3 (5.7)	84.6 (19.4)	.96
No. of BP medications^a^	2.7 (1.2)	2.5 (1.0)	.68
BP measurement			
Submitted ≥1	10 (100.0)	3 (30.0)	.001
No. of measurements^a^	20.9 (10.5)	3.9 (6.6)	<.001

Abbreviations: BP, blood pressure; HFpEF, heart failure with preserved ejection fraction; HFrEF, heart failure with reduced ejection fraction.

^a^ Values are presented as the mean (SD).

Survey responses are shown in Table 2. More patients in the text-based group found it “very easy” to send back measurements compared with patients using the online portal (10 [100.0%] vs 0 [0.0%]; *P* < .001).

**Table 2.** Poststudy Survey Responses Regarding Patient Satisfaction in the Text-Based Message Group and Online Patient Portal Group

Question	No. of patients (%)		*P* value
	Text-based message group (n = 10)	Online patient portal group (n = 10)	
Do you have reliable access to a computer with internet?			
Yes	5 (50.0)	9 (90.0)	.05
Do you have reliable access to a smartphone with internet?			
Yes	9 (90.0)	9 (90.0)	>.99
How satisfied were you with the program?			
Very satisfied	9 (90.0)	5 (50.0)	.14
Somewhat satisfied	1 (10.0)	4 (40.0)	
Neutral		1 (10.0)	
How easy was it to send your BP measurements back to your doctor?			
Very easy	10 (100.0)	0	<.001
Somewhat easy	0	3 (30.0)	
Neither difficult nor easy	0	4 (40.0)	
Difficult	0	2 (20.0)	
Unsure	0	1 (10.0)	
After participating, I feel checking my BP at home is…			
Very easy	10 (100.0)	8 (80.0)	.33
Somewhat easy	0	1 (10.0)	
Neither easy nor difficult	0	1 (10.0)	
How likely are you to recommend this program to a friend?			
Very likely	8 (80.0)	8 (80.0)	>.99
Somewhat likely	2 (20.0)	2 (20.0)	
Are you planning on using the online portal to communicate with your doctors?			
Yes	4 (40.0)	2 (20.0)	.11
Maybe	6 (60.0)	2 (20.0)	
No	0	1 (10.0)	
Unsure	0	3 (30.0)	
Compared to before the program, I plan to check my BP at home…			
More frequently	7 (70.0)	9 (90.0)	.24
The same amount	0	1 (10.0)	
Less frequently	2 (20.0)	0	
Unsure	1 (10.0)	0	
How do you feel about home BP monitoring rather than coming in to see the doctor for a BP visit?			
I prefer home BP monitoring with video visits to follow-up	1 (10.0)	7 (70.0)	.12
I prefer to come to the doctor’s office for BP checks and treatment	9 (90.0)	3 (30.0)	
Is there anything else you would like to tell us about the program?	Program helped me to understand my blood pressure and how to keep it down This program helped me a lot with the cuff to see how important it is to check my pressure daily	Too hard to get online and use the portal. Don’t know how to use phone. Need granddaughter to help My daughter has to enter it for me; it’s too hard for me. I don’t know how myself Son had to put app on the phone for me. Then lost phone and new phone didn’t get the app back on the phone. Hard to do without help; will try to get doctor to reinstall on phone	NA

Abbreviations: BP, blood pressure; NA, not applicable.

## Discussion

As the first study of its kind—to our knowledge—to be conducted during the COVID-19 pandemic, we found higher uptake of and satisfaction with a text-based program compared with an online patient portal for home BP monitoring among Black patients with Medicaid or Medicare insurance, hypertension, and CVD or CVD risk factors. The use of technology to manage chronic diseases may exacerbate disparities in health care; however, the type of technology used is important.^3^ Although enrollment in this study required broadband access, accessing online patient portals may be prohibitive for patients from historically marginalized groups. The digital divide has been well documented; video use for telemedicine visits during the pandemic has been shown to be significantly lower among Black and low-income patients.^3^ In this study, only 3 patients (30.0%) in the online patient portal group engaged with the online portal.

Text-based communication has been shown to be an effective way for patients to engage with providers.^4^ At our institution, a text-based program for BP management among postpartum women was shown to improve patient outcomes and reduce health care disparities.^5^ This pilot trial was limited by the small sample size; therefore, these results should be confirmed in larger studies because text-based care may be an effective way to reach patients who have historically faced barriers to accessing care for chronic conditions.

## Notes

Supplement 1.
Trial Protocol

Supplement 2.
Data Sharing Statement
