# Supplementary material for: Evaluation of Online Patient Portal vs Text-Based Blood Pressure Monitoring Among Black Patients With Medicaid and Medicare Insurance Who Have Hypertension and Cardiovascular Disease
Source: JAMA Netw Open. 2022 Feb 15;5(2):e2144255. doi: 10.1001/jamanetworkopen.2021.44255 (PMC8848204; doi:10.1001/jamanetworkopen.2021.44255)
Supplement: Supplement 1. — Trial Protocol [file jamanetwopen-e2144255-s001.pdf]

Patient-Portal vs Text-based Hypertension Monitoring Among Black Medicaid/Medicare Patients

Original Study Protocol

September 1, 2020

Protocol Outline

1. Abstract

2. Overall objectives

3. Aims

    3.1 Primary outcome

    3.2 Secondary outcomes

4. Background

5. Study design

    5.1 Design

    5.2 Study duration

    5.3 Target population

    5.5 Key inclusion criteria

    5.6 Key exclusion criteria

6. Subject recruitment

7. Subject compensation

8. Study procedures

    8.1 Consent

    8.2 Procedures

9. Analysis plan

10. Investigators

11. Human research protection

    11.1 Data confidentiality

    11.2 Subject confidentiality

    11.3 Subject privacy

11.4 Data disclosure

11.5 Data safety and monitoring

11.6 Risk/benefit

11.6.1 Potential study risks

11.6.2 Potential study benefits

11.6.3 Risk/benefit assessment

12. References

13. Appendix

13.1. Patient Satisfaction Survey

13.2. Automated Blood Pressure Text-Responses

1. Abstract

Black patients suffer a disproportionate burden of uncontrolled hypertension and cardiovascular disease (CVD). Self-measured blood pressure (BP) is associated with improved BP control, especially when combined with telemonitoring. However, the digital divide has been well-documented and disparities in accessing telemedicine may limit the benefit of home BP monitoring for Black patients. The aim of this study was to use a randomized pilot trial to evaluate the uptake and acceptability of a text-based model for home BP monitoring compared to an online-patient portal among low-income Black patients with hypertension and CVD.

2. Overall objectives

The objective of this study is to use a randomized, clinical trial to compare the uptake and satisfaction of a text-based home blood pressure monitoring program, as compared to to the currently available blood pressure monitoring program using the patient portal (My Penn Medicine) that is integrated into the electronic health record (the current standard of care).

3. Aims

3.1 Primary outcome

The primary outcome variable is the number of blood pressure measurements sent to the provider over the 14-day enrollment period between groups (text-based vs. My Penn Medicine).

3.2 Secondary outcomes

Secondary outcomes include the following:

- Proportion of patients who sent >1 blood pressure measurement over the 14 day enrollment period

- Satisfaction of utilization of text-message home blood pressure monitoring as compared to My Penn Medicine
- Systolic blood pressure
- Diastolic blood pressure

4. Background

The coronavirus disease 2019 (COVID-19) pandemic has uprooted conventional health care delivery for routine ambulatory care, requiring health systems to rapidly adopt telemedicine capabilities. The digital divide, has been well documented with lower rates of technology and broadband adoption among racial/ethnic minorities [1-5]. Additionally, Black patients suffer a disproportionate burden of hypertension and cardiovascular disease [6]. This program will

implement a text-based home hypertension monitoring program among Black Medicaid patients with hypertension and cardiovascular disease (CVD) and compare its uptake to the currently available blood pressure monitoring program using the patient portal (My Penn Medicine) that is integrated into the electronic health record (EHR).

5. Study design

5.1 Design

We will conduct a two-arm randomized, controlled trial comparing text-based home blood pressure monitoring, as compared to the current standard of care, which is home blood pressure monitoring and sending in measurements through My Penn Medicine, the EHR-based online portal. Patients will be randomized to one of the two arms. All participants will be provided with a blood pressure cuff at an in-person visit at enrollment, and will be enrolled in a 14-day home blood pressure monitoring program, and instructed to check their blood pressure and send in measurements twice daily. After 14 days of blood pressure monitoring, all participants are sent an exit patient satisfaction survey.

<u>Study population</u>

We will recruit Black patients (based on self-report as documented in the EHR) seen in-person at Hospital of University of Pennsylvania Cardiovascular clinic with Medicaid or Medicare/Medicaid, and ICD 10 diagnosis of hypertension plus one of the following: Coronary artery disease, myocardial infarction, cerebrovascular disease, peripheral arterial disease, heart failure, or diabetes mellitus type 2.

<u>Trial arms</u>

Arm 1. Text-Based Home Blood Pressure Monitoring Program: Individual participants will be instructed to check blood pressure at home and text to a number provided. They will receive automated daily text-reminders and feedback on blood pressure.

Arm 2. EHR –based online portal home blood pressure monitoring program (current standard of care): Per standard of care, individual participants will be instructed to check their blood pressure daily and upload measurements to the My Penn Medicine online portal, either daily or at the end of the 14-day period.

<u>Intervention</u>

Participants in all arms will be given a blood pressure cuff during an in-person visit on enrollment and will be informed of the AHA recommendation guidelines checking blood pressure and receive additional interventions as follows:

1) Arm 1: Text-Based Home Blood Pressure Monitoring Program:
    a. 14-day enrollment period
    b. Sent a 'test' text message upon enrollment in clinic to ensure they have the correct number to text blood pressure measurements
    c. Participants will receive a daily text message reminding them to send their blood pressure measurements as well as automated feedback regarding their blood pressure measurements.
2) Arm 2. EHR –based online portal home blood pressure monitoring program
    a. All participants will undergo the same enrollment period of 14-days.
    b. Patients will be given instructions on how to enroll and use the My Penn Medicine Portal
    c. Patients will be instructed to log onto My Penn Medicine and enter their twice daily blood pressures and dates. Once submitted, these values will be forwarded to the ordering provider.

5.2 Study duration

This is a 14-day study with rolling enrollment beginning in October 2020; patients will be given an additional 14 days after enrollment for follow-up to complete the survey with a total follow-up time of 28 days; data analysis to occur after enrollment.

5.3 Target population

Adult patients age ≥ 18 years seen in-person at Hospital of University of Pennsylvania Cardiovascular clinic with Medicaid or Medicare/Medicaid, and ICD 10 diagnosis of hypertension plus one of the following: coronary artery disease, myocardial infarction, cerebrovascular disease, peripheral arterial disease, heart failure, obesity, hyperlipidemia, or diabetes mellitus type 2. As a feasibility study, we will enroll 20 patients for this pilot study.

5.4 Key inclusion criteria

1) Age ≥18 years;
2) Seen in Hospital of University of Pennsylvania Cardiovascular clinic
3) Diagnosis of chronic hypertension
4) Diagnosis of cardiovascular disease or risk factors to include: coronary artery disease, myocardial infarction, cerebrovascular disease, peripheral arterial disease, heart failure, obesity, hyperlipidemia, or diabetes mellitus type 2.
5) Ability to read and provide informed consent to participate in the study;
6) Has smartphone and broadband access

7) Home blood pressure monitoring is recommended by cardiologist
8) Do not have a blood pressure cuff at home

5.5 Key exclusion criteria

1) Does not speak English
2) Blood Pressure >180/120 on initial check
3) Patients without a phone with texting capabilities or online access (phone or home computer) to enroll in My Penn Medicine

6. Subject recruitment

The PI, Dr. Eberly, will reach out to cardiology consultative providers at the University of Pennsylvania Cardiovascular clinic to let them know about the study and the inclusion/exclusion criteria. Participants will be recruited in clinic at the conclusion of their cardiology visit. After consent is obtained to enroll in the study, patients will be provided with a blood pressure cuff and instructions on study enrollment for the specific arm they are randomized to. Patients enrolled in the text-based arm will receive a 'test' text during the in-person visit, confirming enrollment. After the 14-day enrollment period, all participants will receive a text linking them to an online survey regarding patient satisfaction with the program.

7. Subject compensation

All participants will receive a blood pressure cuff for enrolling.

8. Study procedures

8.1 Consent

If a patient is deemed to potentially meet inclusion criteria in the study by their provider, the PI will be contacted. The PI, Dr. Eberly, will meet with the patient at the end of the in-person visit to discuss the study and obtain verbal consent. We will not ask participants to sign a written consent form, as this would be redundant and create unnecessary paperwork with identifiable information. After consenting, participants in the text-based arm will be sent a 'test' text. Participants will be provided with details regarding how to contact the research team via phone at any time if they subsequently wish to withdraw from the study or have questions.

8.2 Procedures

Potentially eligible participants will be identified from providers at the Hospital of University of Pennsylvania Cardiovascular clinic. The PI, Dr. Eberly, will confirm that those patients meet

eligibility criteria, and if so, she will meet with them at the end of their in-person visit at the cardiology clinic. If the patient does meet criteria and wishing to enroll, at this time, she will obtain verbal consent, as well as instructions for enrollment based on assigned arm.

All participants will receive a blood pressure cuff from the PI, Dr. Eberly. All participants will be asked to use the device to check their blood pressure twice daily for 14 days. Patients in the text-based arm will receive automated daily reminders to check their blood pressure, as well as automated responses based on their blood pressure measurement. If blood pressure is elevated with systolic >180mmHg or diastolic >120 mmHg, patient will be provided to recheck blood pressure in 5 minutes. If blood pressure remains elevated above those parameters, the patient's provider will be notified. Providers will be similarly notified of any blood pressure measurement in that range for patients that send in measurements via the online-portal.

After 14-day period, all blood pressure measurements will be sent to providers. Participants in both groups after the 14-day period, will receive a text with a link to an online survey. Study personnel will abstract clinically relevant data from the electronic medical record to collect data on demographics, clinical comorbidities. The collection of this data will obviate the need to further burden participants by asking them additional questions during the survey periods.

9. Analysis plan

To compare sample characteristics between arms we will use t-tests or Wilcoxon rank-sum tests (F-tests or Kruskal-Wallis test) for continuous variables and Pearson chi square tests or Fisher's exact tests for categorical variables. All hypothesis tests will be two-sided using a two-sided alpha of 0.05 as our threshold for statistical significance. We will use SAS and/or R to analyze the data.

10. Investigators

Lauren Eberly, MD, MPH is the co-Principal Investigator (PI) and is a cardiovascular fellow at the University of Pennsylvania. She currently spends 50% of her effort on research and 50% on clinical activities.

Jennifer Lewey, MD, MPH is the co-Principal Investigator (PI) and is an Assistant Professor of Medicine at the Perelman School of Medicine. She is a faculty member in the Consultative Cardiology Program, Director of the Women's Cardiovascular Health Program, and co-Director of the Pregnancy and Heart Disease program. She currently spends 75% of her effort on research and 25% on clinical and administrative activities.

11. Human research protection

11.1 Data confidentiality

Paper-based records will be kept in a secure location and only be accessible to personnel involved in the study. Computer-based files will only be made available to personnel involved in the study through the use of access privileges and passwords. Wherever feasible, identifiers will be removed from study-related information. Precautions are in place to ensure the data are secure by using passwords and encryption, because the research involves web-based surveys.

11.2 Subject confidentiality

Research material will be obtained from text responses, participant surveys and clinical data abstracted from the electronic health record (EHR). All participants will provide verbal informed consent for access to these materials. The data to be collected include demographic data (e.g., age, sex, self-identified race) and clinical outcome data. Research material that is obtained will be used for research purposes only. The study identification number, and not other identifying information, will be used on all data collection instruments. All study staff will be reminded to appreciate the confidential nature of the data collected and contained in these databases. All data will be stored on password-protected devices hosted on the secure Penn Medicine server. We will implement multiple, redundant protective measures to guarantee the privacy and security of the participant data. All investigators and research staff with direct access to the identifiable data will be required to undergo annual responsible conduct of research, cybersecurity, and HIPAA certification in accordance with University of Pennsylvania regulations. Electronic access rights are carefully controlled by University of Pennsylvania system managers. We believe this multi-layer system of data security, identical to the system protecting the University of Pennsylvania Health Systems medical records, greatly minimizes the risk of loss of privacy. Each subject will be assigned a unique identifier without identifying information, and data will be entered into an electronic database using only the unique identifier. Only trained study staff will have access to the code that links the unique identifier to the subject's identity. Electronic data will be stored on secure, password-protected firewalled servers at the University of Pennsylvania.

11.3 Subject privacy

Enrollment will include a description of the voluntary nature of participation, the study procedures, risks and potential benefits in detail. The enrollment procedure will provide the opportunity for potential participants to ask questions prior to making a decision to participate. Participants will be told that they do not have to answer any questions if they do not wish and can drop out of the study at any time, without affecting their medical care or the cost of their care. All efforts will be made by study staff to ensure subject privacy.

11.4 Data disclosure

No one other than the members of the research team may receive protected health information (PHI) for this research study.

11.5 Data safety and monitoring

At the time of enrollment in the study, all patients are given anticipatory guidance on when to seek medical attention. In addition, as detailed above, for any blood pressure that is significantly elevated or significantly reduced, patient's medical provider will be immediately notified for further instructions.

11.6 Risk/benefit

11.6.1 Potential study risks

This study will allow for patients to monitor their blood pressure at home, which is often utilized by providers to care for patients with hypertension. A potential risk of this study is a breach of participant confidentiality. We will minimize this risk by linking individual identifying information with participant ID numbers only in one single secure file that will only be accessed by the study team in the case of an adverse medical event, participant dropout, or if otherwise deemed necessary by the Principal Investigator. All other identifying information will be discarded after initial contact with the PI, Dr. Eberly. Even the study arms will be identified by code letters until both the statistician and PI agree that analysis is complete.

11.6.2 Potential study benefits

Through participation in this study, each participant will have the potential to better understand how to check their blood pressure at home, and what their blood pressure goals are. Home blood pressure monitoring could improve their health and reduce their risk for future cardiovascular disease or events. This study will also help inform what type of home-blood pressure monitoring program is most acceptable to this group of patients. Participants may also receive no benefit from their participation in the study.

11.6.3 Risk/benefit assessment

Anticipated risks of this study should be minimal and the risk/benefit ratio is very favorable.

12. References

1. National Telecommunications and Information Administration and U.S. Department of Commerce: Falling Through the Net: Defining the Digital Divide. Washington, DC: Government Printing Office, 1999. 14. Chaudhry B., et al.: Systematic review: Impact of health
2. Pew Research Center, April, 2015, "The Smartphone Difference" Available at: http://www.pewinternet.org/2015/04/01/us-smartphone-use-in-2015/. Accessed May 19th, 2020.
3. Pew Research Center, May 2017, "Digital divide persists even as lower-income Americans make gains in tech adoption". Available at: https://www.pewresearch.org/fact-tank/2019/05/07/digital-divide-persists-even-as-lower-income-americans-make-gains-in-tech-adoption/. Accessed May 20th, 2020.
4. Pew Research Center, June 2015, "Americans' Internet Access: 2000-2015". Available at: https://www.pewresearch.org/internet/2015/06/26/americans-internet-access-2000-2015/. Accessed May 20th, 2020.
5. Pew Research Center, April 2020, "53% of Americans Say the Internet Has Been Essential During the COVID-19 Outbreak". Available at: https://www.pewresearch.org/internet/2020/04/30/53-of-americans-say-the-internet-has-been-essential-during-the-covid-19-outbreak/. Accessed May 21, 2020.
6. Benjamin EJ, Virani SS, Callaway CW, et al. Heart disease and stroke statistics-2018 update: a report from the American Heart Association. Circulation. 2018; 137:e67.

13. APPENDIX DOCUMENTS

13.1 POST-COMPLETION SURVEY (Sent via text through survey monkey).

All patients will receive, but those not enrolled in the text-based program will not receive question 4.

1. Prior to this study, I checked my blood pressure at home:
   - Daily or more
   - A few times a week
   - A few times a month
   - Rarely
   - Never
   - Only when I don't feel well

2. During the study, I checked my blood pressure:
   - Twice a day or more
   - Daily
   - A few times a week
   - Weekly
   - Never

3. I found checking my blood pressure at home:
   - Very easy or easy
   - Neither easy nor difficult
   - Very difficult or difficult
   - Unsure

4. I found using the text-message based system for blood pressure monitoring at home:
   - Convenient
   - Neither convenient nor inconvenient
   - Very convenient or convenient

5. I believe that now I will check my blood pressure at home:
   - More frequently
   - The same amount
   - Less frequently
   - Unsure

6. How would you feel about text-based care for blood pressure management rather than coming in to see the doctor?
   - Text-program more convenient/Prefer text-based care instead of coming into the doctor’s office
   - Prefer coming in to the doctor’s office for blood pressure care
   - Would prefer a combination of both

7. Do you have access to a smart phone with internet?
   - Yes
   - No
   - Sometimes
8. Do you have access to a computer with internet?
   - Yes
   - No
   - Sometimes
9. Do you have access to text messaging?
   - Yes
   - No
   - Sometimes

13.2.1 AUTOMATED TEXT RESPONSES

TIMING	TEXT
**Enroll patients. Patients provided BP cuff. Test text**	Welcome to the home blood pressure monitoring program! Please reply to this number with all blood pressure measurements. Thank you!
**Day 1 after enrollment reminder text**	Hello. This is a reminder to text us your blood pressures. What is your blood pressure this morning? Don’t forget to check again in the evening. Thank you!
**Text reminder to check BP everyday**	Good morning! Reminder to keep checking your blood pressure and texting to this number!
**At 2 weeks, congrats text completing study sent**	Congrats on completing the blood pressure program! Your doctor will receive your readings. Thank you! You will receive a survey shortly

13.2.2. AUTOMATED RESPONSES BASED ON BLOOD PRESSURE

**BP normal range (SBP <130 and DBT <80)**	Your blood pressure looks great! Keep up the good work
**BP elevated but systolic <180 and diastolic <120**	Your blood pressure is slightly elevated. Remember to avoid salt in your diet, stay active, and take your medications as recommended. Continue to monitor your blood pressure.
**BP elevated and systolic >180 and/or diastolic >120**	You blood pressure is elevated. Please recheck your blood pressure in 5 minutes and send us the number.
**After 5 min recheck: If systolic >180 and/or diastolic >120**	Thank you for rechecking your repeat blood pressure. We will notify your providers who will be in touch with any updates.
**After 5 min recheck: If systolic <180 and diastolic <120**	Thank you for rechecking your repeat blood pressure. Your blood pressure is elevated. Continue to monitor your blood pressure and take your medications as recommended.
**Any text message not related to blood pressure**	Please only report blood pressure recordings to this number. If you need medical assistance, please call your doctor.
