# Supplementary material for: Evaluation of Online Patient Portal vs Text-Based Blood Pressure Monitoring Among Black Patients With Medicaid and Medicare Insurance Who Have Hypertension and Cardiovascular Disease
Source: JAMA Netw Open. 2022 Feb 15;5(2):e2144255. doi: 10.1001/jamanetworkopen.2021.44255 (PMC8848204; doi:10.1001/jamanetworkopen.2021.44255)
Supplement: Supplement 2. — Data Sharing Statement [file jamanetwopen-e2144255-s002.pdf]

Data Sharing Statement

Eberly. Evaluation of Online Patient Portal vs Text-Based Blood Pressure Monitoring Among Black Patients With Medicaid and Medicare Insurance Who Have Hypertension and Cardiovascular Disease. *JAMA Netw Open*. Published February 15, 2022. doi:10.1001/jamanetworkopen.2021.44255.

Data

Data available: Yes
Data types: Deidentified participant data
How to access data: Deidentified participant data will be made avaliable on reasonable request to the first author, Lauren Eberly, at lauren.eberly@pennmedicine.upenn.edu
When available: With publication

Supporting Documents

Document types: Statistical/analytic code
How to access documents: Statistical/analytic code will be made avaliable on reasonable request to the first author, Lauren Eberly, at lauren.eberly@pennmedicine.upenn.edu
When available: With publication

Additional Information

Who can access the data: Anyone upon reasonable request
Types of analyses: For any research purpose
Mechanisms of data availability: After approval of a proposal
